# Attribution of nosocomial seeding to long-term care facility COVID-19 outbreaks

**DOI:** 10.1017/S0950268823001565

**Published:** 2023-10-25

**Authors:** Joe Flannagan, Dimple Y Chudasama, Russell Hope, Simon M Collin, Alex Bhattacharya, Rachel Merrick, Nurin Abdul Aziz, Susan Hopkins, Gavin Dabrera, Theresa Lamagni

**Affiliations:** 1 United Kingdom Health Security Agency, London, UK; 2 Public Health Wales, Cardiff, UK

**Keywords:** care home, COVID-19, England, long-term care facility, nosocomial

## Abstract

Residents of long-term care facilities (LTCFs) were disproportionately affected by the COVID-19 pandemic. We assessed the extent to which hospital-associated infections contributed to COVID-19 LTCF outbreaks in England. We matched addresses of cases between March 2020 and June 2021 to reference databases to identify LTCF residents. Linkage to health service records identified hospital-associated infections, with the number of days spent in hospital before positive specimen date used to classify these as definite or probable. Of 149,129 cases in LTCF residents during the study period, 3,748 (2.5%) were definite or probable hospital-associated and discharged to an LTCF. Overall, 431 (0.3%) were identified as index cases of potentially nosocomial-seeded outbreaks (2.7% (431/15,797) of all identified LTCF outbreaks). These outbreaks involved 4,521 resident cases and 1,335 deaths, representing 3.0% and 3.6% of all cases and deaths in LTCF residents, respectively. The proportion of outbreaks that were potentially nosocomial-seeded peaked in late June 2020, early December 2020, mid-January 2021, and mid-April 2021. Nosocomial seeding contributed to COVID-19 LTCF outbreaks but is unlikely to have accounted for a substantial proportion. The continued identification of such outbreaks after the implementation of preventative policies highlights the challenges of preventing their occurrence.

## Introduction

In England, 15,536 registered long-term care facilities (LTCFs) offer residential care to an estimated 410,000 people [1]. As these facilities provide extra support for residents, these individuals very often have chronic and complex health needs and are vulnerable to severe consequences from infectious diseases [2]. Such populations are more susceptible to acquiring COVID-19, experiencing severe symptoms, and dying from the disease [3, 4]. LTCF residents in England have been disproportionately affected by the COVID-19 pandemic, constituting over 30% (circa 20,000) of COVID-19 deaths in England in 2020 [5–8]. To help prevent and contain COVID-19 outbreaks in LTCFs, a range of measures were introduced at various time points, including minimising staff movement between facilities, routine resident and staff testing, and preventing or reducing visitors.

Coronaviruses, such as Middle East respiratory syndrome (MERS) and SARS-CoV-2, have the potential to cause high levels of transmission in hospital settings [9, 10]. Whilst a recent UK study estimated 5% of COVID-19 cases to have acquired their infection in the hospital, the subsequent spread in other settings such as LTCFs has to date not been quantified [11]. Given the frequent hospital stays by LTCF residents, reflecting their complex health needs, the seeding of subsequent outbreaks in LTCFs via the transfer of persons with COVID-19 infections acquired in the hospital has been of particular concern [12–14]. Once introduced, there is clear potential for prolific spread due to close proximity and frequent interaction between staff and residents [15].

Our study used national data collected on hospital admissions and COVID-19 cases detected as part of the ongoing pandemic response in England to identify outbreaks in LTCFs potentially seeded by COVID-19 hospital-associated cases. We evaluated the extent of potential nosocomial seeding of LTCF outbreaks in England during the first and second waves of the pandemic and its impact on the overall number of cases and deaths.

## Methods

SARS-CoV-2 was made a notifiable disease in England and Wales on 5 March 2020 [16]. All test-confirmed COVID-19 cases must be reported to the UK Health Security Agency (UKHSA)’s national laboratory reporting system (Second Generation Surveillance System, SGSS) by the National Health Service (NHS) and private laboratories in England [16]. Cases were identified as LTCF residents through residential address matching [7]. The residential address for all positive cases was obtained from SGSS, derived from i) laboratory information management system (LIMS), ii) directly from the case (or their carer) entering it at the time of test registration, or iii) NHS summary care records. The LIMS address, supplied by the diagnosing laboratory, or address entered at the time of testing was utilised preferentially as it should reflect the address at the time of testing, as opposed to the centrally held NHS address, which may not include recent or temporary address changes.

All cases identified among LTCF residents with specimen dates between 1 March 2020 and 26 June 2021 or who were part of LTCF outbreaks that started within this time period were included in this analysis. The data were extracted on 11 August 2021.

### Address matching using the full address

Full addresses in case records were matched against reference databases, namely Ordnance Survey (OS) Care Quality Commission (CQC) list of LTCFs and OS AddressBase Premium database [7]. The former comprises all LTCFs registered with CQC, the national regulator for health and social care, and the latter is populated from local authority databases containing all addresses in England. From these databases, the Unique Property Reference Number (UPRN) was used to group together cases residing at the same property and the associated property use class (Basic Land and Property Unit, BLPU) was used to identify LTCFs.

Environmental Systems Research Institute (ESRI) LocatorHub version six software was used to facilitate the matching of residential addresses to reference databases using a cascaded process starting with exact address matching and then, for records that failed this, additional probabilistic matching steps to allow for minor discrepancies. On the remaining unmatched records, a manual matching process was undertaken. Cases not matched through the aforementioned process were matched by a unique patient identifier (NHS number) to the NHS England Master Patient Index from which the residential address for the case held by their general practice (GP) was obtained.

### Mortality

A COVID-19-associated death was defined as a person with a laboratory-confirmed infection who either died ≤60 days after their first positive specimen date or had COVID-19 mentioned as a main or contributory cause of death on their death certificate [17]. This encompasses deaths in all settings. Mortality information was compiled from the following sources: Office for National Statistics (ONS), NHS England, Local Health Protection Teams (HPTs), and the NHS Spine database.

### Hospital admissions

The NHS Digital Secondary Uses Service (SUS) Admitted Patient Care and Emergency Care Dataset (ECDS) were accessed to identify hospital admissions. Due to delays in reporting, the data were censored six weeks before the date of data extraction.

Hospital records from SUS and ECDS were linked deterministically to case data using NHS number and date of birth, or local hospital patient identifier and date of birth if the former were incomplete. SUS was used to identify recent hospital admissions, defined as a stay encompassing the case’s specimen date or a hospital discharge up to 14 days before the specimen date. ECDS was used to identify cases who attended an Accident and Emergency (A&E) Department and were subsequently admitted to hospital but for whom no SUS data were available; these cases were categorised as having no evidence of discharge. Cases were excluded from further analysis if their most recent hospital record could be categorised under any of the following: an ECDS record from more than three months before extraction that indicated hospital admittance; an ECDS record with a hospital stay of more than one day; and a record with a code indicating discharge but without a discharge date.

### Definitions

#### Outbreak

An outbreak was defined as two or more test-confirmed cases at the same residence (determined by UPRN), within a rolling 14-day window. The case with the earliest specimen date in an outbreak was considered the index case. Where more than one case was diagnosed within the first two days of an outbreak, these were considered ‘co-primaries’.

#### Hospital-associated infections

Cases were grouped according to likely place of acquisition based on the timing of SARS-CoV-2-positive specimen collection and length of hospital admission, using information obtained from SUS and ECDS (Supplementary Figure S1). For the purposes of this analysis, the length of stay was calculated based on overnight stays; if someone was admitted one day and discharged the next, this counted as one day of hospital admission. Hospital-onset hospital-associated (HO·HA) infections were defined as those with a positive test ≥3 days after hospital admission but before hospital discharge. These infections were further categorised into groups based on the likelihood of hospital acquisition. These groups were indeterminate, probable, or definite based on the number of days from hospital admission to the specimen date. Indeterminate, probable, and definite HO·HA infections were defined as a positive test between 3 to 7 days, 8 to 14 days, and ≥ 15 days after admission, respectively (Supplementary Figure S1) [11].

Community-onset hospital-associated (CO·HA) infections were defined as a case with a specimen date not contained within any hospital stay or within the first two days of hospital admission. The case must also have spent at least three days within the hospital between day 1 and day 12 where day 14 is the date of positive specimen. CO·HA cases were divided into subgroups based on how many days between days 1 and 12 were spent within the hospital: indeterminate (3 to 6 days of 12), probable (7 to 11 days of 12), and definite (all 12 days) (Supplementary Figure S1).

#### Potentially nosocomial-seeded outbreak

The following criteria were applied for an LTCF outbreak to be defined as a potentially nosocomial-seeded outbreak: a) the index case of the outbreak must have met a definite or probable hospital-associated case definition and have evidence of discharge to a care home; b) subsequent case(s) were identified with a specimen date between 2 and 14 days (inclusive) after the index case’s specimen date (where specimen date is day 1); and c) the subsequent case(s)’ specimen date must be ≥2 days after the index case’s hospital discharge date. Where multiple cases in an outbreak had identical earliest specimen dates, any case meeting a hospital-associated infection definition with evidence of discharge to a care home was considered the index case for the sake of the analysis.

### Sensitivity analyses

Two separate sensitivity analyses were conducted that expanded the primary analysis to include I) indeterminate hospital- or community-onset cases contributing to potential nosocomial seeding cases of an outbreak and II) co-primary cases that met the criteria to be a potential nosocomial seeding case of an outbreak as described above.

### Interval between infection onset and discharge

Time intervals between specimen and discharge dates for hospital-associated cases were calculated. The calculation of this interval for non-seeding HO·HA cases was limited to those with a specimen date within 14 days before their discharge date to exclude cases that could never have seeded outbreaks. Similarly, non-seeding CO·HA cases were limited to those with a specimen date within seven days following their discharge date.

### Statistical analysis

Stata (StataCorp. 2017. Stata Statistical Software: Release 15. College Station, TX: StataCorp LLC) was used for all analyses.

## Results

### Characteristics of LTCF resident cases and outbreaks

**Figure 1.**
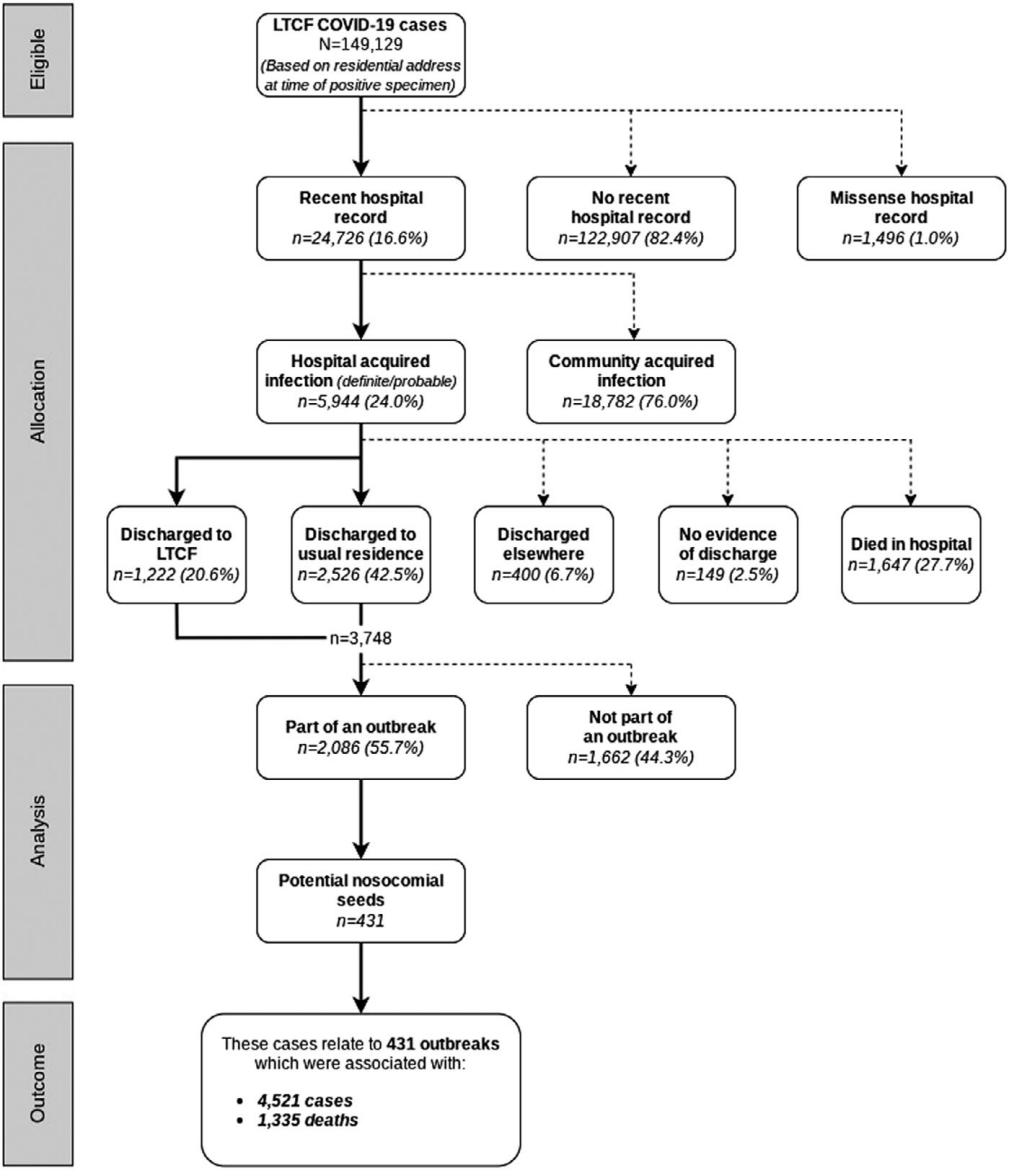
Data flow to identify index cases of potentially nosocomial-seeded COVID-19 outbreaks in LTCFs from all COVID-19 cases between March 2020 and June 2021 in England.

We identified 149,129 COVID-19 cases resident in an LTCF at the time of diagnosis between 1 March 2020 and 26 June 2021, which related to 15,797 outbreaks and 37,178 deaths (Table 1). Of the LTCF residents, 16·6% (n = 24,726) had a specimen date during or within 14 days following a hospital stay (based on the first positive specimen date) (Figure 1). Of these, 24·0% (n = 5,944) met a definite or probable hospital-associated infection definition: 2,177 (36·5%) definite hospital-onset (HO), 2,204 (37·1%) probable HO, 218 (3·7%) definite community-onset (CO), and 1,350 (22·7%) probable CO (Table 2). Among the 5,944 LTCF residents with definite or probable hospital-associated infection, 63·1% (n = 3,748) were identified as having been discharged back to an LTCF (Table 2). A total of 431 ensuing outbreaks were identified as potentially seeded by one of these hospital-associated (HA) (nosocomial) cases discharged to an LTCF. This represents 2·7% (431/15,797) of all LTCF outbreaks, involving 4,521 cases in residents (3·0% of all LTCF cases) (Table 1). Of these outbreaks, 43 (10·0%) were categorised as definite HO·HA, 73 (16·9%) as probable HO·HA, 34 (7·9%) as definite CO·HA, and 281 (65·2%) as probable CO·HA. The median number of cases involved in these outbreaks was 6 (IQR: 3–15), with the largest consisting of 54 cases. The median outbreak length (time between the first and last cases) was 14 (IQR: 8–22) days with the longest lasting 65 days. A total of 1,335 deaths were identified among these outbreaks, representing 3·6% (1,335/37,178) of all COVID-19 deaths in LTCF residents over this period.

**Table 1.** Characteristics of COVID-19 cases and outbreaks in LTCFs in England between March 2020 and June 2021

	All LTCF cases	Index cases of potentially nosocomial-seeded LTCF outbreaks^a^	All cases in potentially nosocomial-seeded LTCF outbreaks
*Overview*			
LTCF resident cases	149,129	..	4,521 (3·0%)
LTCF resident deaths	37,178	..	1,335 (3·6%)
LTCF outbreaks	15,797	..	431 (2·7%)
Median size (cases)	4 (IQR: 2–11)	..	6 (IQR: 3–15)
Median length (days)^b^	8 (IQR: 3–16)	..	14 (IQR: 8–22)
*Age (years)*			
Median (IQR)	83 (69–90)	85 (77–90)	85 (76–90)
*Sex*			
Male	51,174 (34·3%)	184 (42·7%)	1,435 (31·7%)
Female	97,857 (65·7%)	247 (57·3%)	3,085 (68·3%)
*Region*			
East Midlands	15,406 (10·3%)	60 (13·9%)	743 (16·4%)
East of England	17,967 (12·1%)	68 (15·8%)	750 (16·6%)
London	11,417 (7·7%)	30 (7·0%)	263 (5·8%)
North East	9,551 (6·4%)	22 (5·1%)	141 (3·1%)
North West	23,062 (15·5%)	59 (13·7%)	686 (15·2%)
South East	26,519 (17·8%)	54 (12·5%)	708 (15·7%)
South West	12,892 (8·6%)	24 (5·6%)	267 (5·9%)
West Midlands	17,125 (11·5%)	57 (13·2%)	509 (11·3%)
Yorkshire and Humber	15,138 (10·2%)	57 (13·2%)	454 (10·0%)
*Ethnicity*			
Asian	3,412 (2·3%)	5 (1·2%)	64 (1·4%)
Black	3,629 (2·4%)	6 (1·4%)	74 (1·6%)
Mixed	676 (0·5%)	1 (0·2%)	10 (0·2%)
Other	752 (0·5%)	1 (0·2%)	16 (0·4%)
Unknown	7,334 (4·9%)	2 (0·5%)	220 (4·9%)
White	133,326 (89·4%)	416 (96·5%)	4,137 (91·5%)

a Using the definite and probable hospital-associated definitions for the index cases.

b Excludes outbreaks ongoing at the time of data extraction.

**Table 2.** Breakdown by definitions of hospital-associated COVID-19 cases residing in LTCFs in England between March 2020 and June 2021

	No. of LTCF resident cases	No. of LTCF residents discharged to LTCFs	No. of potential nosocomial seeds of outbreaks	No. of cases associated with potentially nosocomial-seeded outbreaks	No. of deaths associated with potentially nosocomial-seeded outbreaks
Definite HO·HA	2,172 (18·0%)	1,250 (15·3%)	43 (4·2%)	256 (2·2%)	83 (2·5%)
Probable HO·HA	2,204 (18·2%)	1,216 (14·9%)	73 (7·0%)	749 (6·6%)	223 (6·8%)
Indeterminate HO·HA	2,699 (22·4%)	1,550 (19·0%)	66 (6·4%)	690 (6·1%)	211 (6·5%)
Total HO·HA	7,075	4,016	182	1,695	517
Definite CO·HA	218 (1·8%)	182 (2·2%)	34 (3·3%)	371 (3·3%)	113 (3·5%)
Probable CO·HA	1,350 (11·2%)	1,100 (13·5%)	281 (27·1%)	3,145 (27·6%)	916 (28·0%)
Indeterminate CO·HA	3,428 (28·4%)	2,863 (35·1%)	539 (52·0%)	6,189 (54·3%)	1,724 (52·7%)
Total CO·HA	4,996	4,145	854	9,705	2,753
Total	12,071	8,161	1,036	11,400	3,270

Abbreviations: CO·HA, community-onset hospital-associated; HO·HA, hospital-onset hospital-associated.

### Sensitivity analyses

With the inclusion of outbreaks potentially seeded by indeterminate hospital-associated cases (both hospital- and community-onset), the number of outbreaks rose to 1,036 (6·6% of all LTCF outbreaks). This encompassed 11,400 (7·6%) cases and 3,270 (8·8%) deaths.

Restricting analyses to probable and definite hospital-associated cases but including co-primary cases as potential seeds, namely a hospital-associated case being one of a number diagnosed within the first two days of an outbreak but not necessarily the first, the proportion of potentially nosocomial-seeded outbreaks increased to 3·6% (n = 565) of all LTCF outbreaks in the study period.

### Temporal trends in outbreaks and deaths

**Figure 2.**
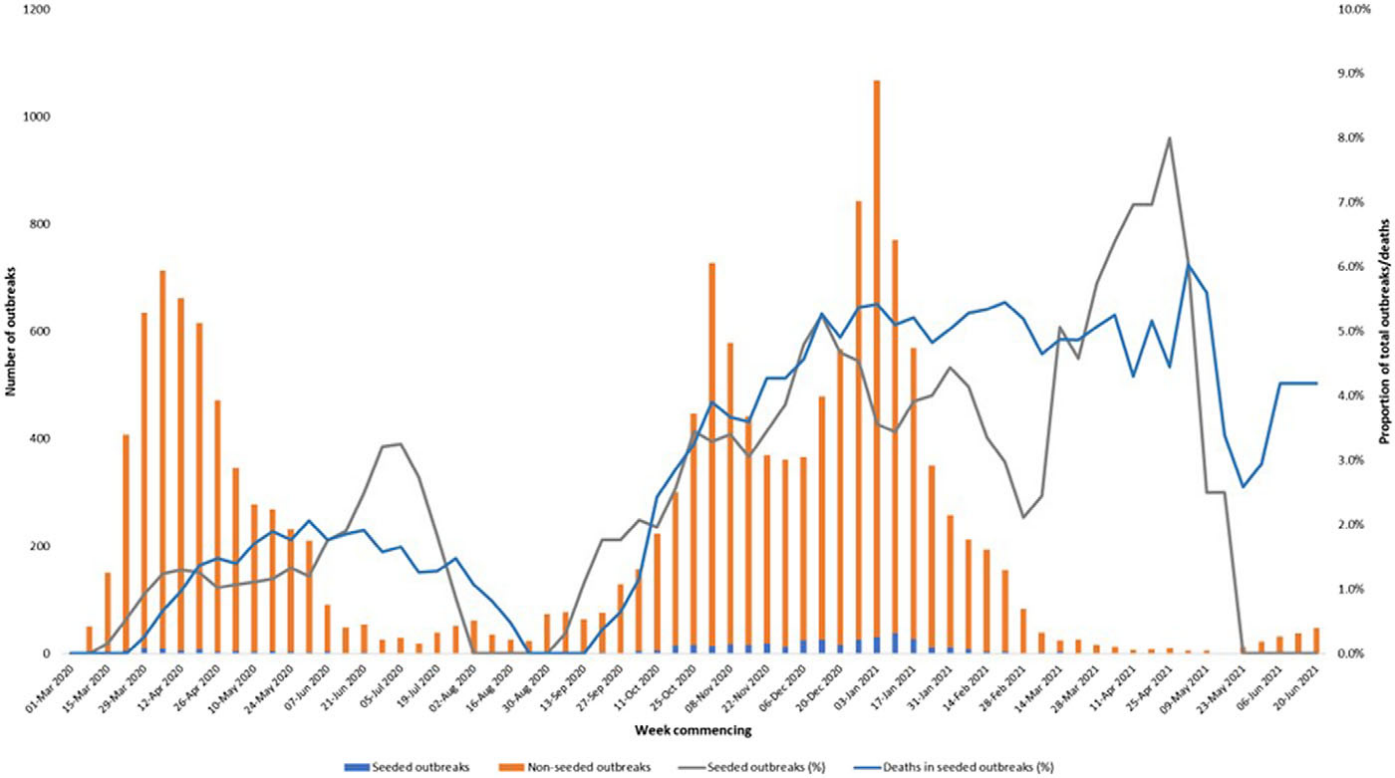
Weekly number of COVID-19 outbreaks in LTCFs between March 2020 and June 2021 in England split by those potentially seeded by a nosocomial case and those not, the proportion of outbreaks in LTCFs identified as potentially nosocomial-seeded outbreaks (monthly rolling average), and the proportion of deaths associated with potentially nosocomial-seeded outbreaks (monthly rolling average). Outbreaks are grouped into weeks based on the specimen date of the first case and deaths are grouped into weeks based on the date of death.

The proportion of deaths in LTCF residents associated with nosocomial-seeded outbreaks increased from March 2020 until mid-June 2020 and then declined before rising again from September 2020 to a peak of 6·5% (77/1,179) in the week commencing 27 December 2020. Based on dates when outbreaks started (specimen week of first case), the proportion of potentially nosocomial-seeded outbreaks peaked in late June 2020 (3·8%), 12 weeks after the first pandemic peak; early December 2020 (6·8%), five weeks after the second pandemic peak; mid-January 2021 (4·9%), one week after the third pandemic peak; and mid-April 2021 (14·3%), a period of very low numbers of outbreaks (Figure 2).

Additionally, the proportion of LTCF outbreaks that were potentially nosocomial-seeded was 1.3% (68/5,265) before regular testing for residents was rolled out on 6 July 2020. From this date to the end of our study period, this proportion was 3.4% (363/10,532).

### Timing of diagnosis for hospital-associated cases

Of the 5,944 LTCF residents with a definite or probable hospital-associated infection, 73.6% (n = 4,376) first tested positive whilst in the hospital, but the majority of the index cases of potentially nosocomial-seeded outbreaks were individuals diagnosed after discharge (73.1%; n = 315).

**Figure 3.**
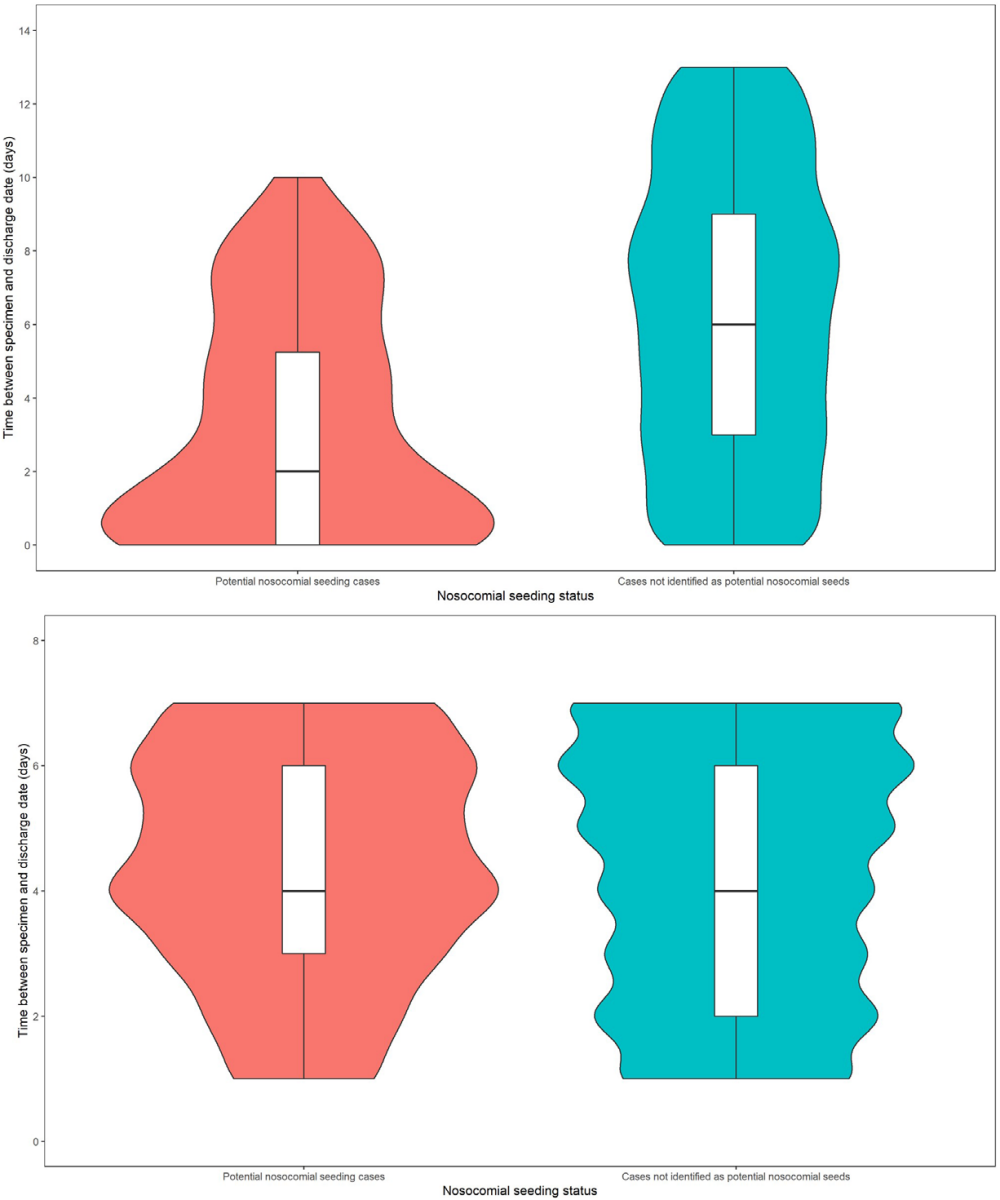
Violin plots showing the distribution of time between specimen date and discharge date for hospital-associated (probable and definite) cases identified as potential nosocomial seeds of LTCF outbreaks and hospital-associated cases not identified as potential nosocomial seeds between March 2020 and June 2021 in England, split by hospital-onset infection (a) and community-onset infection (b).

The median time interval between specimen and discharge dates for HO·HA cases identified as potential nosocomial seeds was 2 (IQR 0–5·5) days before discharge, shorter than for HO·HA cases not seeding outbreaks (median 6 days, IQR 3–9) (Figure 3a). The median time from discharge to diagnosis for CO·HA cases identified as potential nosocomial seeds was 4 (IQR 3–6) days, the same as for CO·HA cases not identified as potential seeds (4 days; IQR 2–6) (Figure 3b).

## Discussion

This is the first comprehensive study in England to assess the role of COVID-19 nosocomial seeding events in LTCF outbreaks and to quantify the impact on subsequent cases and deaths. Our findings suggest that nosocomial seeding contributed to a small proportion of COVID-19 outbreaks, cases, and deaths in LTCFs in England. Our findings are consistent with others conducted in Wales and Scotland that found nosocomial-seeded outbreaks did not account for the majority of LTCF outbreaks in those countries [18–20].

The continued occurrence of outbreaks potentially seeded by a nosocomial case after the implementation of preventative policies highlights the challenge of preventing these outbreaks. Many COVID-19 prevention and control policies were introduced during and after the first wave; for example, in April 2020, guidance was published to test all patients before discharge to an LTCF, and in June 2020, guidance was published to isolate all LTCF residents discharged from hospitals for 14 days. It is not possible to assess the impact of these policies from the data we present, although we note that whilst most hospital-associated COVID-19 infections in LTCF residents were diagnosed during hospital admission, the majority of the outbreaks potentially seeded by them were linked to cases diagnosed after discharge. This difference may suggest a degree of outbreak prevention as a result of the policies in place.

Four peaks were seen in the proportion of outbreaks potentially started by a nosocomial seed, the first three of which occurred in the wake of major peaks in the pandemic. This suggests a rise in the incidence of COVID-19 nationally, and corresponding hospital admissions, may have increased risk of nosocomial seeding in LTCFs. Alternative documented routes of COVID-19 incursions to LTCFs have included staff, visitors, and healthcare professionals [20–22]. Indeed, through our approach we found proportionally few outbreaks among LTCF residents to be potentially nosocomial-seeded, indicating that the majority of outbreaks are not explained by nosocomial seeding alone and that other pathways of infection introduction were more significant. Multiple researchers have considered other pathways of infection and suggested that staff are potentially a key source of infection, particularly those who were asymptomatic and/or who moved between LTCFs [20–22]. As visiting was stopped or heavily restricted across most of the study period, visitors were unlikely to have been a significant contributor to the LTCF outbreaks identified.

Several studies suggest that the prevalence of COVID-19 in the community is the most important factor influencing LTCF outbreaks. Vaccination programmes in these facilities and in the wider community may therefore have the most significant impact on LTCF outbreaks [20, 23]. Research also suggests that the size of LTCFs is positively correlated with COVID-19 outbreaks, a likely reflection of the higher number of residents and staff to bring the infection into the setting [19, 20]. Research into design features to limit transmission in larger facilities may therefore be important to reduce infection levels, as well as for longer-term policy considerations.

COVID-19 vaccine rollout commenced in England in December 2020 with LTCF residents and staff among those in the first priority group for vaccination. A target to offer all LTCF residents and staff a first dose was met on 31 January 2021. By mid-February, over 90% of residents had received at least one dose, but by June 2021 only 65% of facilities met the recommended minimum vaccination level for staff (80% having had at least one dose). This will have reduced overall case and outbreak numbers we identified from December 2020 onwards. We were not able to quantify this potential differential impact in our study.

Whilst our study presents the most comprehensive assessment of nosocomial seeding in England to date, it has limitations due to the data that were available. Primarily, without genomic information for the majority of LTCF resident cases, we cannot confirm transmission from one case to another and instead rely on the alignment of likely windows of transmission, either from hospital settings to inpatients or from discharged patients to other LTCF residents. With the more recent expansion of genomic sequencing, with specific targeting of LTCFs, such a study may become possible during periods where a mix of genotypes is circulating. However, data linkage approaches like ours are well established in England and can still provide valuable epidemiological information [24, 25]. Secondly, our study relies on case ascertainment by testing. From 6 July 2020, whole LTCF testing was introduced on a monthly basis for residents, thus limiting the impact of missed cases. However, before this time, which includes the majority of wave 1, testing capacity was limited. For this reason, it is likely that our estimate for the number of outbreaks potentially seeded by a nosocomial case in wave 1 represents an underestimate, as well as the number of cases and deaths linked to such outbreaks. This could explain the disparity between the proportions of all outbreaks that were potentially nosocomial-seeded before and after 6 July 2020 (1.3% vs. 3.4%). It is therefore impossible to know whether the peak in potentially nosocomial-seeded outbreaks identified in June 2020 is a true peak, or rather the result of data on cases and outbreaks before this being underestimated. Thirdly, our address-matching methodology means that we were unable to identify cases in LTCF staff as they do not reside in the facility. This means both the size and, to a lesser extent, the number of LTCF outbreaks may be underestimated and that we were unable to assess the contribution of this alternative route of COVID-19 ingress. Furthermore, the identification of LTCF residents relied primarily on the address entered at the time of test. Thus, individuals admitted to the hospital from a private residence who tested positive within their hospital stay and were subsequently discharged to an LTCF would not be identified as residents of that facility.

Overall, our study provides a critical and revealing look at potential nosocomial seeding of COVID-19 outbreaks in LTCFs using individual data linkage for all LTCF resident cases covering the full pandemic up until June 2021. Understanding the routes of transmission in this setting is vitally important to our understanding of the pandemic in England and of COVID-19 infection prevention going forward.

## Supporting information

Flannagan et al. supplementary material 1 Flannagan et al. supplementary material

Flannagan et al. supplementary material 2 Flannagan et al. supplementary material

## Data Availability

The data that support these studies were collected as part of a public health response and are considered sensitive and not made publicly available. Reasonable requests for access to anonymised data and data dictionary will be considered by the authors on request to allow all results to be reproduced.

## Supplementary material

The supplementary material for this article can be found at https://doi.org/10.1017/S0950268823001565.
