# Supplementary figures and images for: Attribution of nosocomial seeding to long-term care facility COVID-19 outbreaks
Source: Epidemiol Infect. 2023 Oct 25;151:e191. doi: 10.1017/S0950268823001565 (PMC10728972; doi:10.1017/S0950268823001565)

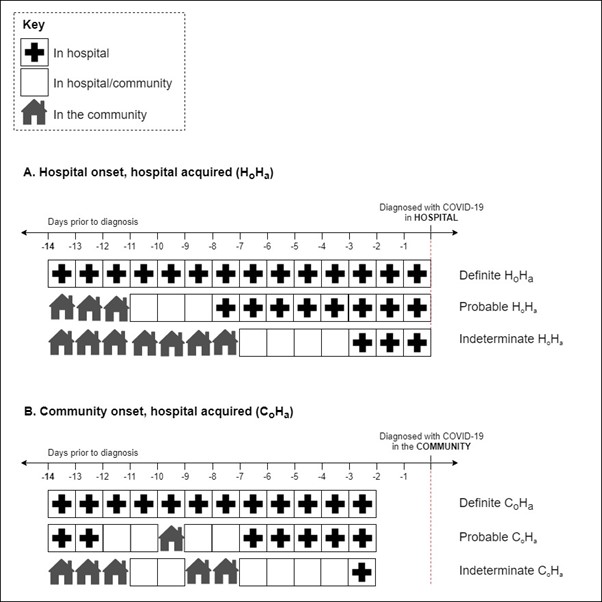

Supplement: Flannagan et al. supplementary material 1 — Flannagan et al. supplementary material [file S0950268823001565sup001.jpg]

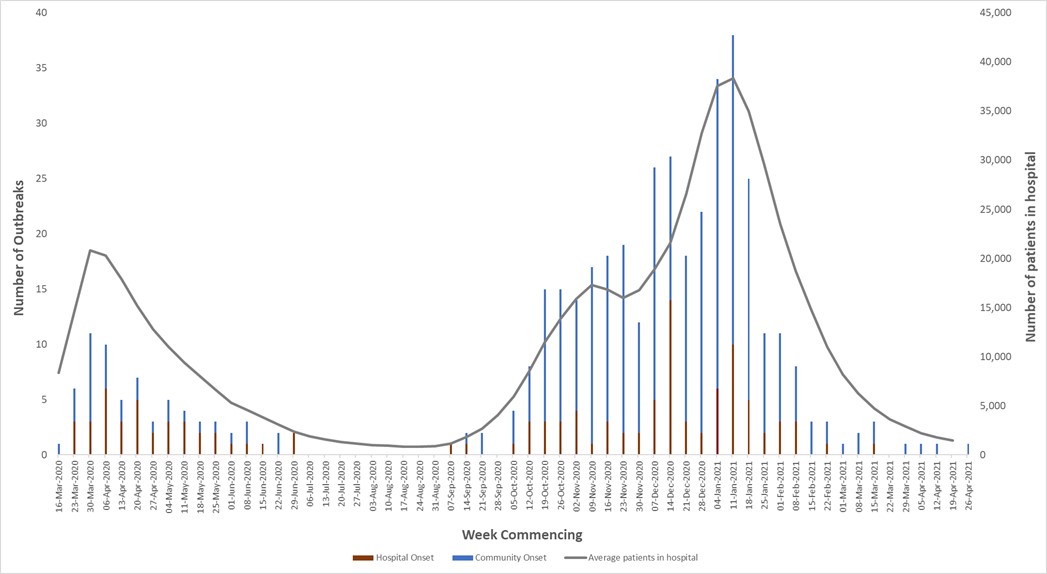

Supplement: Flannagan et al. supplementary material 2 — Flannagan et al. supplementary material [file S0950268823001565sup002.jpg]
